# Elevated CO_2_ Drives the Enrichment of Multidrug Resistance Genes in Paddy Soils

**DOI:** 10.3390/toxics14060467

**Published:** 2026-05-26

**Authors:** Fen Xu, Qian Xiang, Guobing Wang, Xitian Peng, Youxiang Zhou, Hongyan Guo

**Affiliations:** 1 Hubei Key Laboratory of Nutritional Quality and Safety of Agro-Products, Institute of Agricultural Quality Standards and Testing Technology Research, Hubei Academy of Agricultural Sciences, Wuhan 430064, China; 2 Key Laboratory of Urban Environment and Health, Institute of Urban Environment, Chinese Academy of Sciences, Xiamen 361021, China; 3 Zhejiang Key Laboratory of Urban Environmental Processes and Pollution Control, CAS Haixi Industrial Technology Innovation Center in Beilun, Ningbo 315830, China; 4 Institute of Geographical Sciences, Henan Academy of Sciences, Zhengzhou 450046, China; 5 State Key Laboratory of Pollution Control and Resource Reuse, School of the Environment, Nanjing University, Nanjing 210023, China

**Keywords:** antibiotic resistance genes, FACE system, soil bacterial communities, paddy soil

## Abstract

Antibiotic resistance genes (ARGs) are becoming a global issue due to the emergence of superbugs. However, the impact of elevated CO_2_ (eCO_2_) on the soil antibiotic resistome remains largely unknown. Here, using a free-air CO_2_ enrichment platform, we employed high-throughput quantitative PCR and 16S rRNA gene sequencing to investigate the effect of eCO_2_ (ambient + 200 ppm) on soil ARGs and bacterial communities in a paddy ecosystem at harvest. The results showed that eCO_2_ had no significant effect on rice biomass. A LEfSe analysis identified a clear taxonomic shift, with taxa such as c_Clostridia, *g_Dehalobacter* and *g_Syntrophus* being significantly enriched under eCO_2_. The total relative abundance of ARGs increased 1.5-fold under eCO_2_, driven by a 2.8-fold increase in multidrug resistance genes. The correlation and network analyses revealed that the proliferation of specific potential host bacteria was the primary driver of the observed ARG enrichment under eCO_2_. Together, this study offers new insights into the eCO_2_-driven alterations of soil antibiotic resistomes, highlighting the elevated dissemination potential of multidrug resistance genes within paddy ecosystems and their potential implications for food safety.

## 1. Introduction

Climate change and antibiotic resistance are converging threats to global health [1,2]. As a primary driver of climate change, elevated atmospheric carbon dioxide (eCO_2_) alters plant–soil interactions by modifying plant carbon assimilation and subsequent root exudation [3,4]. Given that the soil microbiome is a major reservoir of antibiotic resistance genes (ARGs) and is exquisitely sensitive to carbon availability [5,6], it is imperative to determine how the antibiotic resistome will respond to this fundamental global change.

Atmospheric CO_2_ levels have risen steadily over the past century, surpassing 420 ppm in recent years, and are projected to continue increasing due to anthropogenic activities [7,8]. Increases in the atmospheric CO_2_ concentration may impact soil microbial communities, which are essential drivers of plant growth and ecosystem health [9]. Specifically, enhanced photosynthesis under eCO_2_ increases the flux of labile carbon into the rhizosphere via root exudation, fundamentally reshaping ecological selection pressures [10]. While the effects on microbial richness (alpha diversity) are variable, this resource affects community composition across diverse ecosystems [9,11]. This process often favors fast-growing, copiotrophic taxa (e.g., phyla Bacteroidetes, Proteobacteria) that can rapidly capitalize on these new resources [11,12,13]. Given that the soil microbiome is the primary reservoir of ARGs [14], this predictable, plant-driven shift in the host community composition necessitates a critical re-evaluation of the soil resistome’s stability and potential risk under future climate scenarios.

ARGs are widely recognized as emerging contaminants, with soil serving as a major environmental reservoir [15]. The collective ARG repertoire in environmental compartments, termed the environmental resistome, functions as a critical interface between environmental and clinical antibiotic resistance [16,17]. The dissemination of these environmental ARG reservoirs through agroecosystems poses a distinct toxicological relevance, with potential consequences for food safety and human exposure via the soil–plant–food chain. While vertical gene transfer ensures the stable inheritance of ARGs within lineages, the rapid, cross-species dissemination of ARGs is primarily driven by horizontal gene transfer. This process is mediated by mobile genetic elements (MGEs), such as plasmids, transposons, and integrons [18]. Notably, environmental stressors, including heavy metals, and climate factors can co-select for antibiotic resistance through shared genetic mechanisms (e.g., multidrug efflux pumps, integrons) or cross-resistance pathways [19]. Studies have shown that eCO_2_ can not only promote the conjugation and transfer of ARGs by regulating cell surface characteristics and plasmid transfer-related genes [20] but also might reshape the soil antibiotic resistome by modulating the microbial community via changes in plant–soil interactions and biogeochemical cycles [21]. However, the net effect of eCO_2_ on the soil ARGs remains ambiguous, with existing studies presenting a complex picture. For instance, previous studies suggested that eCO_2_ can inhibit the proliferation of specific ARGs under organic fertilization or sulfadiazine pressure [21,22]. In contrast, Qiu et al. [23] found that eCO_2_ did not exert a significant impact on the abundance of efflux pumps genes. This complexity is further amplified by co-contaminants. For example, in a multi-antibiotic contaminated paddy soil, eCO_2_ decreased sulfonamide and tetracycline resistance genes while simultaneously increasing multidrug resistance genes [24]. Furthermore, eCO_2_ has been reported to enrich the relative abundance of seed ARGs and MGEs under conventional agricultural management [25]. Nevertheless, there is limited information about how eCO_2_ independently modulates the soil antibiotic resistome in conventionally managed agricultural ecosystems, hindering our ability to accurately assess future environmental health risks.

Rice paddy ecosystems are crucial to the food security of more than half of the world’s population. In this study, we conducted a field-based pot experiment using the China FACE (free-air CO_2_ enrichment) platform to investigate the effects of elevated CO_2_ on rice productivity, soil physicochemical properties, microbial communities, and soil ARG profiles under chemical fertilizer application. Specifically, we aimed to (1) assess the variation of soil ARGs under eCO_2_; (2) determine the impact of eCO_2_ on soil bacterial community, rice growth and soil properties; and (3) explore the potential main factors shaping soil ARGs under eCO_2_.

## 2. Materials and Methods

### 2.1. FACE System and Pot Experiment

The experiment was performed at the FACE platform situated in Zongcun village, Jiangsu Province, China (119°42′ E, 32°35′ N). The local climate is subtropical monsoonal, averaging 980 mm precipitation and 15 °C annually. The platform design, which has been described previously [26,27], included three FACE plots (eCO_2_, aCO_2_ + 200 ppm) and three ambient control pots (aCO_2_, ~390 ppm, reflecting the current local concentration). Each eCO_2_ plot was encircled by a 12.5 m diameter octagonal tube that released pure CO_2_ gas over the rice canopy during the growth period. The aCO_2_ plots received no CO_2_ supplementation and were separated from the FACE plots by a distance of 90 m. The CO_2_ release was modulated dynamically according to real-time wind direction and speed data, maintaining a stable elevated concentration of ~200 ppm above ambient levels.

The soil utilized in this pot experiment was identified as Shajiang–Aquci–Cambosols, collected from the upper 20 cm of the adjacent agricultural land. Its properties are detailed in Table S1. The collected soil was prepared by air-drying and then grinding it to a particle size of less than 5 mm. Each pot was filled with 4 kg soil. Rice seedlings (*Oryza sativa* L. cv. Wuyunjing 23) were transplanted into each pot on 22 June and harvested at the end of October 2017. Chemical fertilizer (N-P_2_O_5_-K_2_O = 15-15-15) was applied at the following stages: 50% pre-transplanting, 25% tillering, and 25% heading. The total nitrogen content was 22.5 g m^−2^. The water management was conducted in accordance with local rice cultivation practices. Briefly, all pots were kept submerged with a water depth of ~3 cm at the seedling stage. Manual wet–dry cycles were implemented from the late tillering stage to the early jointing stage. During this period, soil was allowed to dry naturally, and a ~3 cm water layer was replenished only upon the appearance of soil micro-fissures. Then all pots were re-submerged to maintain the initial flooding level until ten days prior to harvest. The experimental pots were assigned randomly and evenly distributed in different rings. The rice biomass produced by each pot was determined at harvest. Soil samples were simultaneously collected from each pot and stored at 4 °C for subsequent analysis.

### 2.2. DNA Extraction and High-Throughput qPCR

Total DNA was extracted from fresh soil samples using a Fast DNA Spin Kit (MP Biomedicals, Santa Ana, CA, USA) and quantified using a NanoDrop ND-1000 spectrophotometer (Wilmington, Waltham, MA, USA). The extracted DNA was stored at −80 °C for subsequent analysis.

Using the SmartChip Real-Time PCR system, a high-throughput quantitative PCR (HT-qPCR) analysis was conducted to quantify the abundance of ARGs and MGEs [14,28]. The 296 primers (285 ARGs, 10 MGEs and one 16S rRNA gene) and operational protocol were identical to those described by Xiang et al. [29]. Triplicate technical repeats and negative controls were utilized in the HT-qPCR assays. Only primers meeting the 90–110% efficiency benchmark were retained. A gene was considered validly detected only when it showed amplification signals in all three replicates with a threshold cycle value below 31 [30]. ARG copy numbers were normalized to 16S rRNA gene abundance to account for variations in microbial biomass.

### 2.3. Bacterial 16S rRNA Gene Sequencing

Amplification of the bacterial 16S rRNA gene’s V4–V5 domain was achieved using the primers 515F/907R [28]. Purified PCR products were then sequenced on the Illumina MiSeq 300 platform at Majorbio (Shanghai, China). Sequencing data were processed using QIIME (v1.9.1), and operational taxonomic units (OTUs) were clustered at 97% similarity [31,32]. This OTU-based clustering approach was deployed to facilitate direct and robust comparisons with previous historical data generated from long-term global change and FACE experimental platforms. Taxonomy was assigned using the SILVA database. The resulting OTU table was used to analyze community composition and calculate alpha and beta diversity indices. All sequencing data are available in the NCBI SRA database under accession number PRJNA758632 (SRR15686267-268, 270-279, 282-284).

### 2.4. Soil Physicochemical Properties Analyses

To determine the physicochemical properties, all soil samples were prepared by air-drying and sieving through a 2 mm mesh. The soil pH was measured in a 1:2.5 soil-to-water suspension. Total nitrogen and total phosphorus were quantified using the Kjeldahl and molybdenum-blue colorimetry methods, respectively [33,34]. The concentrations of copper, zinc, lead, and nickel were determined by flame atomic absorption spectrometry (Hitachi Z-2000, Hitachi, Tokyo, Japan) after digestion with HNO_3_-HF-HClO_4_ (5:3:3, *v*/*v*/*v*) [21]. These metals were selected for their established roles in co-selection of antibiotic resistance [19,35].

### 2.5. Statistical Analysis

IBM SPSS Statistics v26 was used to calculate data averages and standard deviations. *T*-tests (*p* < 0.05) were used to evaluate differences among samples. Linear discriminant analysis effect size (LEfSe) was applied to identify biomarkers between different treatments [36]. The LDA score threshold was set at 2.0, and the Kruskal–Wallis test *p*-value threshold was 0.05. The ARG distribution was visualized using Krona Charts [37]. The composition of ARG subtypes, along with their relationships to bacterial taxa, was illustrated through heatmaps produced with the aid of the R package “pheatmap”. The network analyses were performed to explore the co-occurrence patterns (Spearman’s correlation coefficient |r| > 0. 8 with *p* < 0.01) between ARGs and bacterial taxa (OTUs with relative abundance > 0.1%) using R (v4.1.0) with psych package and were then visualized by Gephi 0.9.2 software.

## 3. Results and Discussion

### 3.1. Rice Biomass and Soil Physicochemical Properties Subsection

The aboveground biomass and grain biomass in all the pots ranged from 57.0 to 63.4 g and from 34.5 to 37.2 g, respectively (Table 1). Although eCO_2_ concentrations can enhance rice biomass and yield via the CO_2_ fertilization effect [38,39], the magnitude of this response varies significantly among the different cultivars [40]. In this study, eCO_2_ led to a non-significant increase in the aboveground biomass, grain biomass, and plant height of the rice. This finding was consistent with Wang et al. [41], who reported no significant eCO_2_ effect on rice grain yield, aboveground biomass, or harvest index. The response of the rice growth to eCO_2_ was modulated by a variety of factors, such as the cultivars used, the developmental stage, the nitrogen availability and the temperature [39]. A meta-analysis of 20 years of FACE data revealed that eCO_2_ reduced head rice percentage by 8%, which led to no increase in head rice yield [39]. The japonica rice cultivar was adopted in this study. Previous studies have shown that japonica rice cultivars exhibit relatively weak responses in growth and yield under elevated CO_2_ [40], which may be related to its lower nitrate absorption activity [42]. An elevated CO_2_ concentration slightly decreased the soil pH value, but the difference was not significant. In addition, no significant difference was observed in the soil concentrations of total nitrogen, total phosphorus, copper, zinc, lead and nickel between the aCO_2_ and eCO_2_ conditions. The non-significant changes in soil nutrients and trace elements may partly explain the non-significant difference in rice biomass.

### 3.2. Soils ARGs and MGEs

**Figure 1:**
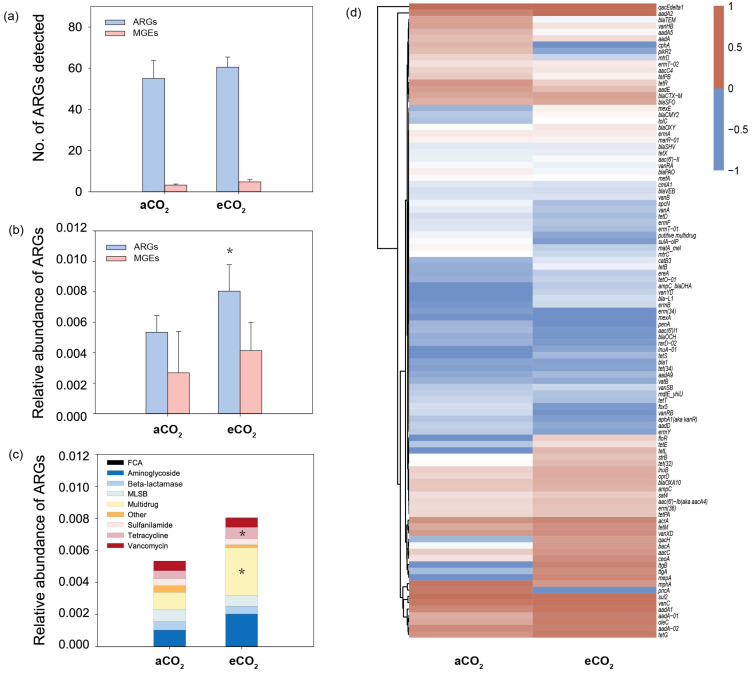
Influence of elevated CO_2_ on soil resistome. (**a**) ARG number, (**b**) relative abundance of total ARGs and (**c**) different ARG types, and (**d**) heatmap of ARG subtypes in soils under ambient (aCO_2_) and elevated CO_2_ (eCO_2_) levels. *, *p* < 0.05.

Through a high-throughput qPCR analysis, an average total of 54 ARGs and 4 MGEs were detected in all the soil samples (Table S2). The eCO_2_ had no significant effect on the number of soil ARGs and MGEs, nor on the abundance of MGEs (Figure 1a). The relative abundance of the detected ARGs in the ambient and elevated treatments was 0.0053 copies/16sRNA and 0.0080 copies/16sRNA, respectively (Figure 1b). The elevated CO_2_ significantly increased the relative abundance of soil ARGs, i.e., 1.5 times higher than that in ambient treatment (Figure 1b).

**Figure 2:**
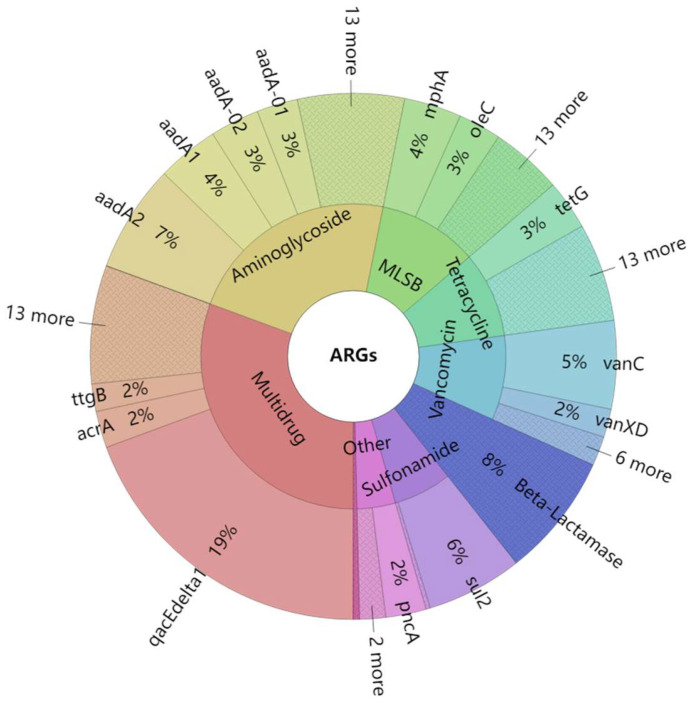
Krona plot of ARGs in all soil treatments.

Multidrug and aminoglycoside resistance genes were the most abundant detected ARG types among all the soil samples (Figure 1c,d). Compared to ambient, eCO_2_ increased the relative abundance of multidrug and tetracycline resistance genes by 2.8 and 1.4 times, respectively (Figure 1c). As shown in Figure 1d, eCO_2_ diminished the relative abundance of *blaTEM* and *vanHB* but enriched *qacEdelta1* and *tetG*. Notably, *qacEdelta1* was the most abundant gene detected in all the soil samples, accounting for 19.4% of the total ARG abundance (Figure 2). The *qacEdelta1* is a significant marker for multidrug resistance. The enrichment of *qacEdelta1* may be a major contributor to the overall rise in ARG abundance under eCO_2_. The influence of eCO_2_ on the soil antibiotic resistome may be associated with variations in CO_2_ exposure duration, pollutants, and potential microbial hosts in the soil. For instance, eCO_2_ enriched multidrug resistance genes in soils contaminated with multiple antibiotics [24]. In contrast, Xu et al. [21] observed that eCO_2_ had no significant effect on the abundance of multidrug resistance genes in both chemical fertilized soils and sulfadiazine-contaminated soils. Our study adds a critical dimension, revealing that even under conventional chemical fertilization without direct antibiotic inputs, eCO_2_ exerted selection pressure on the soil ARGs, particularly the multidrug resistance genes. Moreover, Li et al. [25] found that eCO_2_ can also enrich ARGs and MGEs in rice seeds. Collectively, these findings suggest the net effect of eCO_2_ on the background soil resistome may masked or even reversed by dominant local variables, including soil chemistry and specific selective pressures. From a One Health perspective, the enrichment of multidrug resistance genes under eCO_2_ is linked to a larger soil ARG reservoir, raising potential food safety concerns for agricultural ecosystems under climate change.

### 3.3. Soil Bacterial Communities

**Figure 3:**
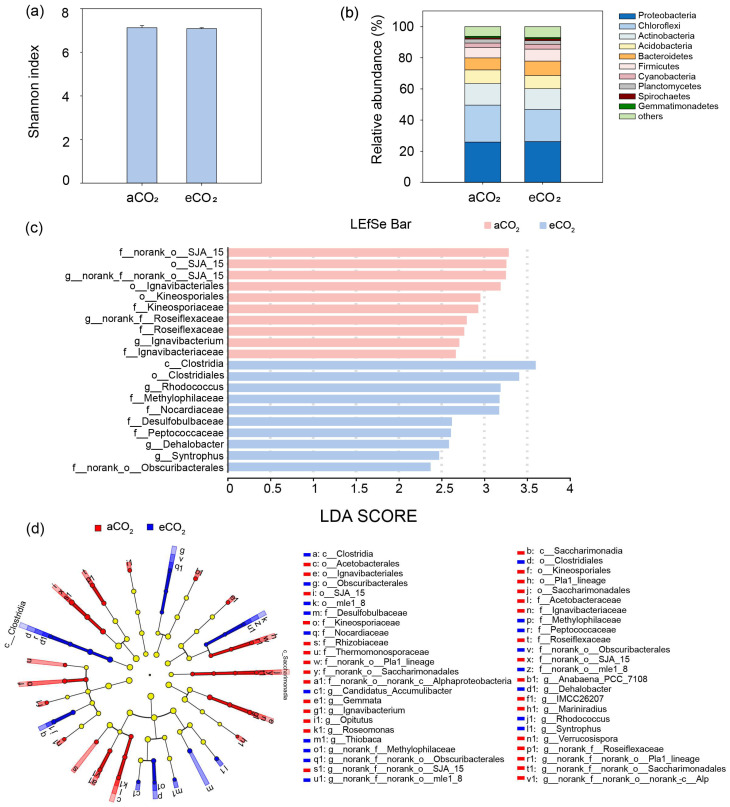
Influence of elevated CO_2_ on soil microbial community composition. (**a**) Shannon index and (**b**) phylum composition of the soil bacterial community, (**c**) LDA analysis indicating the difference biomarkers of taxa and (**d**) cladogram generated by linear discriminant analysis of effect size (LefSe) analysis under different CO_2_ levels.

Elevated CO_2_ did not significantly alter the Shannon index of the soil bacterial community (Figure 3a). At the phylum level, the community was dominated by Proteobacteria, Chloroflexi and Actinobacteria, which collectively constituted 56.7–65.5% of the relative abundance (Figure 3b). The LEfse analysis identified distinct taxonomic biomarkers for each treatment. Specifically, the aCO_2_ treatment was enriched in c_Saccharimonadia, o_Ignavibacteriales, o_Kineosporiales and f_Roseiflexaceae (Figure 3c,d). The dominance of these taxa aligns with oligotrophic microbial ecological strategies, indicating an adaptation to resource-limited conditions. In contrast, it is widely established that eCO_2_ stimulates plant photosynthesis and the consequent inputs of rhizospheric carbon [13,26,43]. This altered resource availability presumably functioned as an agent of environmental filtration, reshaping the ecological assembly through deterministic ecological selection mechanisms that favor copiotrophic lineages capable of utilizing enhanced root exudates. The taxa enriched under eCO_2_ included c_Clostridia, o_Clostridiales, f_Methylophilaceae, f_Nocardiaceae, f_Desulfobulbaceae, f_Peptococcaceae, *g_Dehalobacter* and *g_Syntrophus* (Figure 3c,d). As dominant members of the paddy soil bacterial community, o_Clostridiales act as primary fermenters, breaking down complex polysaccharides into simpler compounds. Through synergistic partnerships with other microorganisms, *g_Syntrophus* achieves the catabolism of complex organic substrates. This process yields simple end-products, notably acetate and formate [44,45]. Furthermore, the members of f_Methylophilaceae rely on methylotrophic metabolism, characterized by the utilization of methanol and methylamine as substrates for energy generation [46]. f_Desulfobulbaceae are important sulfate-reducing bacteria with roles in organic matter degradation and nutrient cycling [47]. Collectively, these findings suggest that elevated CO_2_ may accelerate the decomposition and turnover of plant-derived soil carbon.

### 3.4. Association of Soil ARGs with Bacterial Communities

**Figure 4:**
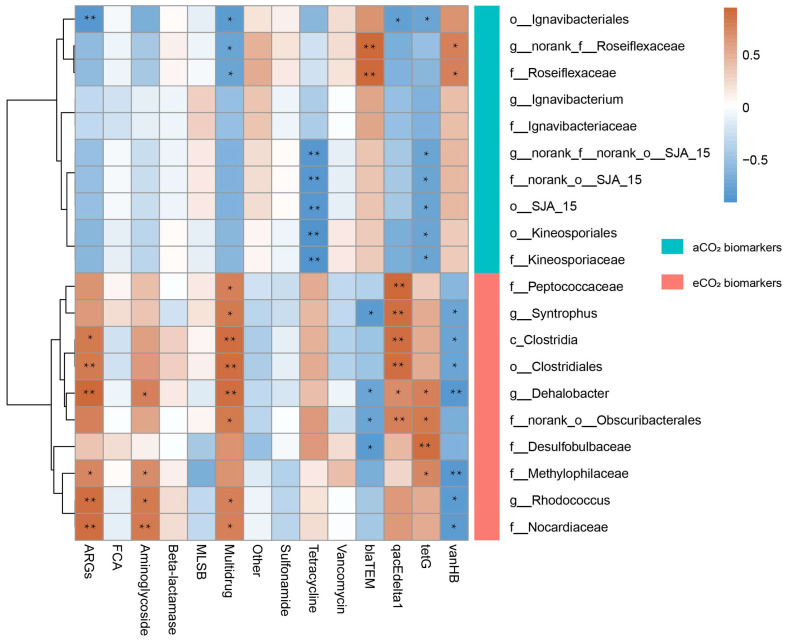
Pearson correlation analysis between microbial biomarkers and ARGs. *, *p* < 0.05; **, *p* < 0.01.

Previous studies have established that the effect of eCO_2_ on ARGs is not direct, but it is driven by plant-mediated rhizosphere effects [21,22,24]. This process reshapes the soil carbon-to-nitrogen stoichiometry and the microbial community composition, thereby regulating the abundance of ARG host bacteria and MGEs, which in turn determines the abundance and horizontal transfer potential of ARGs. The proliferation of ARGs is intrinsically linked to the population dynamics of their host bacteria [48]. A Pearson correlation analysis was employed to explore the association between microbial biomarkers and ARGs (Figure 4). The results showed that the microbial biomarkers enriched under eCO_2_ were strongly associated with an increased prevalence of ARGs. Specifically, the relative abundances of total ARGs, multidrug ARGs, and tetracycline ARGs were significantly positively correlated with eCO_2_-enriched taxa such as c_Clostridia, *g_Rhodococcus*, f_Nocardiaceae, and *g_Dehalobacter*. Conversely, these ARG categories were negatively correlated with the biomarkers characteristic of the ambient CO_2_ treatment, such as f_Kineosporiaceae and o_SJA_15. Previous studies reported that the class Clostridia and its subordinates were ARGs hosts [49,50]. These results suggest that the ecological shift driven by eCO_2_ favors a community with a higher ARGs load.

**Figure 5:**
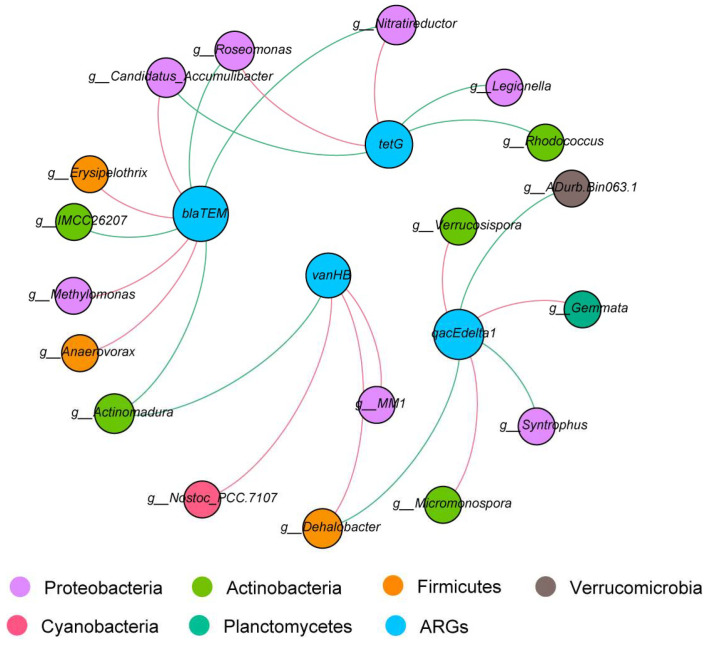
Network analysis between significantly changed ARG subtypes and bacterial genera (with relative abundances >0.01%, excluding taxonomically unclassified groups).

To further explore the potential microbial hosts of ARGs, a network analysis was performed between the significantly changed ARG subtypes and the bacterial genera (with relative abundances >0.01%, excluding the taxonomically unclassified groups) [48]. A total of 18 bacterial genera were significantly correlated with these changed ARG subtypes (Figure 5). Notably, several genera that were enriched under eCO_2_ exhibited positive correlations with specific ARGs (Figure S1), highlighting their potential role as resistance hosts. For instance, *qacEdelta1* was positively linked to the eCO_2_-associated genera *g_Dehalobacter* and *g_Syntrophus* (Figure 5 and Figure S1). *g_Dehalobacter* can act as a host for multiple ARGs in environmental microbial communities [51]. Similarly, *tetG* was strongly associated with the eCO_2_ biomarker *g_Rhodococcus* (Figure 5). Members of *g_Rhodococcus* have been specifically identified as hosts for various ARGs, including those conferring resistance to multiple antibiotics such as aminoglycosides, beta-lactams, tetracyclines, vancomycin, and multidrug resistance genes [52]. In addition, *g_Candidatus_Accumulibacter*, whose abundance was higher under eCO_2_ than that under aCO_2_ (Figure S1), was positively correlated with *tetG* but negatively with *blaTEM* (Figure 5). A previous study observed that *g_Candidatus_Accumulibacter* was positively linked to tetracycline resistance genes in the biological nutrient removal process [53]. *g_Roseomonas* was positively associated with *blaTEM* but negatively with *tetG*, which were significantly decreased under eCO_2_ (Figure 5 and Figure S1). The *bla* gene (β-lactamase) is specifically mentioned as present in Roseomonas genomospecies 5, conferring resistance to β-lactam antibiotics [54]. As the detected abundance of MGEs remained unchanged, the proliferation of specific potential host bacteria may be the primary driver of the observed ARG enrichment under eCO_2_. Additionally, eCO_2_ may promote the increase of ARGs by intensifying the antagonistic relationships between fungi and bacteria [25]. Although elevated CO_2_ induced no dramatic shifts in crop biomass, it may cause effects on plant carbon and nitrogen metabolism and belowground carbon allocation, thereby impacting soil microbial communities and their associated ARGs [55,56]. It is worth noting that due to the intrinsic ecological uncertainty of open soil systems and the potential confounding influence of unmeasured variables, these correlation-based links inherently represent shared ecological niches rather than a definitive genetic alignment [57]. Therefore, these connected bacterial taxa are rigorously interpreted as potential hosts rather than confirmed physical carriers. Further efforts using more soil types at a larger temporal scale using various metagenomics surveys will provide a better understanding of the response and mechanisms of ARG transmission under future climate change scenarios.

## 4. Conclusions

In summary, elevated CO_2_ significantly increases the abundance of ARGs in fertilized paddy soil, despite having no significant effect on rice biomass. This ARG proliferation was primarily driven by a 2.8-fold increase in multidrug resistance genes. The correlation and network analyses revealed that the ARG enrichment was driven by a distinct shift in the soil bacterial biomarkers. Specifically, eCO_2_ selectively promoted the proliferation of potential ARG hosts such as Clostridia, *Rhodococcus*, and *Dehalobacter*. These findings underscore the potential amplification risk of soil multidrug resistance genes under rising CO_2_ levels. The spread of antimicrobial resistance in agroecosystems under climate change may extend the threats to global food safety, emphasizing the necessity of ARG assessment in plants and grains. Incorporating mitigation strategies for these dissemination risks into future agricultural policies is essential to protect global public health.

## Figures and Tables

**Table 1:** Rice growth parameters and soil properties under ambient and elevated CO_2_ levels. Letters a indicate statistically significant differences at *p* ≤ 0.05.

		Ambient CO_2_	Elevated CO_2_
Rice	Aboveground biomass (g pot^−1^)	57.0 ± 2.4 a	63.4 ± 5.1 a
	Grain weight (g pot^−1^)	34.5 ± 2.1 a	37.2 ± 2.6 a
	Tiller no.	11.0 ± 0.4 a	12.5 ± 1.2 a
	Height (cm)	61.8 ± 2.8 a	64.0 ± 2.9 a
Soil	pH	5.69 ± 0.07 a	5.57 ± 0.13 a
	Total nitrogen (g kg^−1^)	1.20 ± 0.05 a	1.17 ± 0.02 a
	Total phosphorus (g kg^−1^)	0.90 ± 0.02 a	0.91 ± 0.04 a
	Zinc (mg kg^−1^)	77.5 ± 0.8 a	82.3 ± 1.5 a
	Copper (mg kg^−1^)	23.3 ± 0.3 a	24.0 ± 0.4 a
	Lead (mg kg^−1^)	31.5 ± 0.2 a	30.5 ± 0.6 a
	Nickel (mg kg^−1^)	18.7 ± 0.5 a	18.8 ± 0.5 a

## Data Availability

The original contributions presented in this study are included in the Article/Supplementary Materials. Further inquiries can be directed to the corresponding author.

## Supplementary Materials

The following supporting information can be downloaded at: https://www.mdpi.com/article/10.3390/toxics14060467/s1, Figure S1: Comparison of bacterial genera that related to significantly changed ARG subtypes between ambient (A) and elevated CO_2_ (F) treatments; Table S1: Physical and chemical properties of soil; Table S2: List of detected ARGs in the soil.
