# Supplementary material for: Elevated CO2 Drives the Enrichment of Multidrug Resistance Genes in Paddy Soils
Source: Toxics. 2026 May 26;14(6):467. doi: 10.3390/toxics14060467 (PMC13306802; doi:10.3390/toxics14060467)
Supplement: Supplementary file 1 [file toxics-14-00467-s001.zip › toxics-4274970-supplementary.pdf]

Elevated CO_2 Drives the Enrichment of Multidrug Resistance Genes in Paddy Soils

Fen Xu [1], Qian Xiang [2, 3], Guobing Wang [4], Xitian Peng [1], Youxiang Zhou [1], Hongyan Guo [5,*]

[1] Hubei Key Laboratory of Nutritional Quality and Safety of Agro-products, Institute of Agricultural Quality Standards and Testing Technology Research, Hubei Academy of Agricultural Sciences/, Wuhan 430064, China;
[2] Key Laboratory of Urban Environment and Health, Institute of Urban Environment, Chinese Academy of Sciences, Xiamen 361021, China;
[3] Zhejiang Key Laboratory of Urban Environmental Processes and Pollution Control, CAS Haixi Industrial Technology Innovation Center in Beilun, Ningbo 315830, China;
[4] Institute of Geographical Sciences, Henan Academy of Sciences, Zhengzhou 450046, China
[5] State Key Laboratory of Pollution Control and Resource Reuse, School of the Environment, Nanjing University, Nanjing 210023, China
* Correspondence: hyguo@nju.edu.cn (H. G.)

Table S1 Physical and chemical properties of soil

	Sand	Silt	Clay	pH	SOM	TN
Soil	57.8%	28.5%	13.7%	6.4	18.6 g kg^{-1}	1.11 g kg^{-1}

Table S2. List of detected ARGs in the soil

Gene name	classification	Mechanism
catb3	(flor)/(chlor)/(am)phenicol	antibiotic deactivate
cml_e1	(flor)/(chlor)/(am)phenicol	efflux pump
aac6ib	Aminoglycoside	antibiotic deactivate
ant2ia	Aminoglycoside	antibiotic deactivate
aac3iv	Aminoglycoside	antibiotic deactivate
ant3ia	Aminoglycoside	antibiotic deactivate
aadA5	Aminoglycoside	antibiotic deactivate
ant6ia	Aminoglycoside	antibiotic deactivate
ant3ia	Aminoglycoside	antibiotic deactivate
ant3ia	Aminoglycoside	antibiotic deactivate
spcN	Aminoglycoside	antibiotic deactivate
aph3iiia	Aminoglycoside	antibiotic deactivate
aac6ib	Aminoglycoside	antibiotic deactivate
aac3vi	Aminoglycoside	antibiotic deactivate
aph6id	Aminoglycoside	antibiotic deactivate
aph3ia	Aminoglycoside	antibiotic deactivate
aac6ia	Aminoglycoside	antibiotic deactivate
aad9	Aminoglycoside	antibiotic deactivate
aadd	Aminoglycoside	antibiotic deactivate

bl2d-oxa10	Beta-Lactamase	antibiotic deactivate
bl2b-tem1	Beta-Lactamase	antibiotic deactivate
bl2be-ctxm	Beta-Lactamase	antibiotic deactivate
bl2be-shv2	Beta-Lactamase	antibiotic deactivate
bl1-sm	Beta-Lactamase	antibiotic deactivate
bl1-pao	Beta-Lactamase	antibiotic deactivate
bl1-cmy2	Beta-Lactamase	antibiotic deactivate
bl2be-oxy1	Beta-Lactamase	antibiotic deactivate
blaSFO	Beta-Lactamase	antibiotic deactivate
bl2-veb	Beta-Lactamase	antibiotic deactivate
bl3-cpha	Beta-Lactamase	antibiotic deactivate
bl1-ampc/dha	Beta-Lactamase	antibiotic deactivate
bl2a-iii	Beta-Lactamase	antibiotic deactivate
bl3-I	Beta-Lactamase	antibiotic deactivate
bl1-och	Beta-Lactamase	antibiotic deactivate
blaTLA	Beta-Lactamase	antibiotic deactivate
fox5	Beta-Lactamase	antibiotic deactivate
lnub	MLSB	antibiotic deactivate
mphA	MLSB	antibiotic deactivate
erea	MLSB	antibiotic deactivate
vatB	MLSB	antibiotic deactivate

ermf	MLSB	cellular protection
erma	MLSB	cellular protection
ermt	MLSB	cellular protection
pikR2	MLSB	cellular protection
erm36	MLSB	cellular protection
ermb	MLSB	cellular protection
ermy	MLSB	cellular protection
ermt	MLSB	cellular protection
mefa	MLSB	efflux pump
matA/mel	MLSB	efflux pump
oleC	MLSB	efflux pump
qacEdelta1	Multidrug	efflux pump
acrA	Multidrug	efflux pump
marR	Multidrug	efflux pump
mtrC	Multidrug	efflux pump
tolc	Multidrug	efflux pump
mtrD	Multidrug	efflux pump
oprd	Multidrug	efflux pump
ceoA	Multidrug	efflux pump
mexe	Multidrug	efflux pump
ttgA	Multidrug	efflux pump

mepA	Multidrug	efflux pump
qacH	Multidrug	efflux pump
rarD	Multidrug	efflux pump
ttgB	Multidrug	efflux pump
mdtE/yhiU	Multidrug	efflux pump
putitive multidrug	Multidrug	efflux pump
baca	other/bacitracin	antibiotic deactivate
pncA	other/Pyrazinamide	other/unknown
sat	other/streptothricin	antibiotic deactivate
dfra1	Sulfonamide	antibiotic deactivate
sul2	Sulfonamide	cellular protection
sulA/folP	Sulfonamide	cellular protection
tetm	Tetracycline	cellular protection
tetpb	Tetracycline	cellular protection
tet32	Tetracycline	cellular protection
tett	Tetracycline	cellular protection
teto	Tetracycline	cellular protection
tets	Tetracycline	cellular protection
tetg	Tetracycline	efflux pump
tetpa	Tetracycline	efflux pump
tetR	Tetracycline	efflux pump

tete	Tetracycline	efflux pump
tetb	Tetracycline	efflux pump
tetd	Tetracycline	efflux pump
tetx	Tetracycline	other/unknown
tet34	Tetracycline	other/unknown
vanxd	Vancomycin	cellular protection
vanhb	Vancomycin	cellular protection
vanra	Vancomycin	cellular protection
vanC	Vancomycin	cellular protection
vansb	Vancomycin	cellular protection
vana	Vancomycin	cellular protection
vanb	Vancomycin	cellular protection
vanrb	Vancomycin	cellular protection

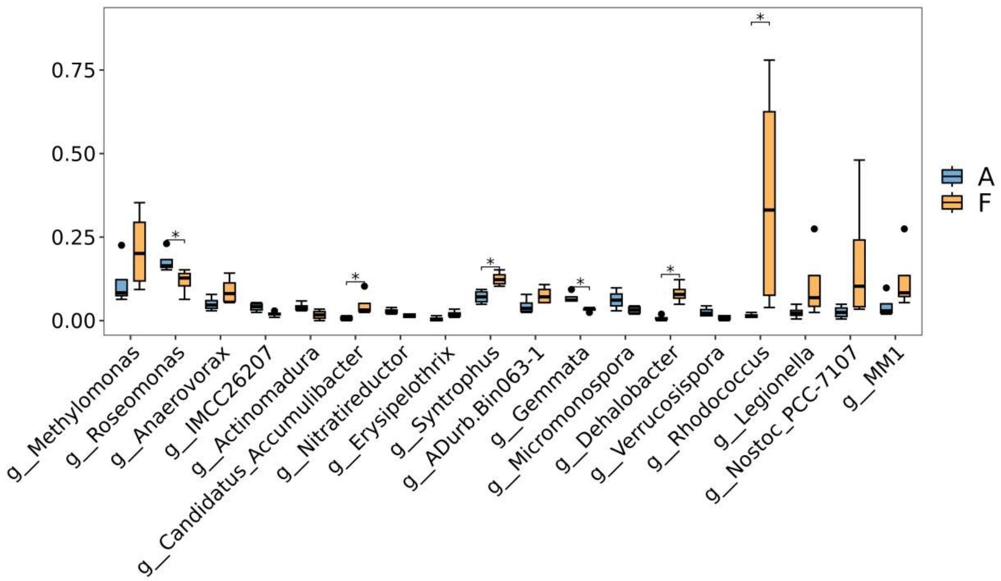

Fig. S1. Comparison of bacterial genera that related to significantly changed ARG subtypes between ambient (A) and elevated CO_2 (F) treatments.
